# A Bio-Inspired Approach to Improve the Toughness of Brittle Bast Fibre-Reinforced Composites Using Cellulose Acetate Foils

**DOI:** 10.3390/biomimetics9030131

**Published:** 2024-02-21

**Authors:** Nina Graupner, Jörg Müssig

**Affiliations:** HSB—Hochschule Bremen, the Biological Materials Group, Department of Biomimetics, Neustadtswall 30, D-28199 Bremen, Germany; jmuessig@bionik.hs-bremen.de

**Keywords:** toughness, impact strength, glass sponge spicule, Hexactinellida, impact modifier, hybrid composite, *Hibiscus cannabinus* L.

## Abstract

Bast fibre-reinforced plastics are characterised by good strength and stiffness but are often brittle due to the stiff and less ductile fibres. This study uses a biomimetic approach to improve impact strength. Based on the structure of the spicules of a deep-sea glass sponge, in which hard layers of bioglass alternate with soft layers of proteins, the toughness of kenaf/epoxy composites was significantly improved by a multilayer structure of kenaf and cellulose acetate (CA) foils as impact modifiers. Due to the alternating structure, cracks are deflected, and toughness is improved. One to five CA foils were stacked with kenaf layers and processed to composite plates with bio-based epoxy resin by compression moulding. Results have shown a significant improvement in toughness using CA foils due to increased crack propagation. The unnotched Charpy impact strength increased from 9.0 kJ/m^2^ of the pure kenaf/epoxy composite to 36.3 kJ/m^2^ for the sample containing five CA foils. The tensile and flexural strength ranged from 74 to 81 MPa and 112 to 125 MPa, respectively. The tensile modulus reached values between 9100 and 10,600 MPa, and the flexural modulus ranged between 7200 and 8100 MPa. The results demonstrate the successful implementation of an abstract transfer of biological role models to improve the toughness of brittle bast fibre-reinforced plastics.

## 1. Introduction

Bast fibres such as kenaf, obtained from the plant stalk, generally result in good strength and stiffness characteristics when used in composites [1]. Toughness, however, is limited due to the low ductility of the fibres [2]. Kenaf (*Hibiscus cannabinus* L.) [3] is widely used for door panels and other interior trim units in automotive applications [4] and many other structural applications [5,6]. The quality of a fibre-reinforced composite is significantly influenced by the processing method, the matrix used, the fibre content, the semi-finished product used (like multilayer fibre web or needle felt, fabric, short fibres or roving), the fibre quality itself, and other criteria [5,6,7,8]. Applications that require higher toughness are often limited due to the brittleness of bast fibres such as kenaf [1]. Therefore, different methods are used to increase the toughness of bast fibre-reinforced composites and to expand their possible application areas. For example, adhesion promoters [9,10] or hybridisation with tougher fibres are used for this purpose. Hybridisation is often applied to improve properties, such as the toughness of a composite. Various approaches exist in the literature; for example, bast and glass fibres or bast and carbon fibres are combined to bring together the favourable properties of both fibre types. The combination of bast and glass fibres, for example, significantly improves toughness and strength compared to pure bast fibre-reinforced composites and leads to better damping characteristics than pure glass fibre-reinforced composites [11,12,13,14]. An overview of the hybridisation of kenaf with different kinds of fibres for composite applications can be found in [15,16].

In recent years, more and more bio-inspired and biomimetic approaches have been used to improve specific mechanical properties of fibre-reinforced composites [17,18,19]. Biological models offer many possibilities and examples of how technical materials can be improved through structural design and material selection with certain properties, e.g., in terms of their toughness [20]. It was possible to develop hybrid materials with significantly improved toughness properties, e.g., based on the model of the coconut fruit shell where stiff and strong layers of bast fibres enclose a core of tougher lyocell fibres (regenerated cellulose). The impact strength of the bio-inspired material with a fibre mass content of 15% bast fibres and 15% lyocell fibres in a polylactide (PLA) matrix could be increased almost to the level of a pure lyocell composite without losing strength and stiffness [21]. In these approaches, the arrangement of the fibre layers with different properties is decisive. It has been shown that stiffer fibres on the outside of the material, which enclose a core of fibres with lower stiffness and higher toughness, have a more beneficial effect on improving toughness than the reverse layer structure [22]. Other approaches to improve the toughness of composite materials can be found, e.g., following the microstructure of the dactylus club of the giant mantis shrimp (Crustacea: Stomatopoda). In this case, the so-called Bouligand structure (a structure consisting of different rotated layers, each having a specific fibre orientation angle) is transferred. The dactylus club consists of the impact surface, the impact region and the periodic region, which are characterised by different properties. The abstract transfer to technical fibre-reinforced composites mainly makes use of the helicoidal structure with specific fibre orientations and the utilisation of materials with different properties. This structural, abstracted design could considerably improve the toughness of many fibre-reinforced composite applications [20,23,24,25].

Numerous other strategies can be found in nature as a source of inspiration for improving the toughness of materials. As an overall principle, improved toughness is often based on crack deflection and crack enlargement in the material so that it does not directly fracture under force. Increasing the crack length leads to increased energy absorption and, thus, to improved toughness [20]. This can be achieved by combining hard (brittle) and soft phases (ductile), e.g., in the form of “brick and mortar” [20]. A well-known biological role model for this type of failure is nacre, which has already been a source of inspiration for improving the toughness of a wide range of materials [26,27,28]. Nacre is tougher than many industrially manufactured ceramic materials. Nacre contains 95 vol. % CaCO_3_ (hexagonal aragonite platelets) with a width of 10–20 µm and a thickness of 0.5 µm, which are arranged in parallel lamellae and can have a work of fracture up multiple times higher than pure aragonite. The platelets are surrounded by a 5% soft organic proteinaceous matrix. When the nacre structure fails, the crack must run around the platelets rather than through them, increasing the length of the crack path and resulting in a higher work of fracture [29]. The structure has been exploited in various ways to improve the toughness of fibre composites, for example, for the development of strong and stiff carbon ‘super nanotubes’ that could be embedded in a softer polymer matrix [30] or a multilayer laminate composite of discontinuous carbon fibre layers and continuous glass fibre layers [31]. Another development was to laser-cut a tiled micro-structure with high accuracy and repeatability at the micro-scale (tiles of the order of 600 mm) onto thin-ply carbon/epoxy prepregs following the nacre model [32].

A similar crack-branching strategy is found in the structure of spicules from the deep-sea glass sponge (Hexactinellida). The skeleton of the glass sponge *Euplectella aspergillum* is complexly composed of hierarchical fibre-like structures (spicules) [33] and, due to its complex structure, can serve as a source of inspiration for constructive materials [34,35,36]. The anchor spicules serve to anchor the glass sponge to the seafloor and consist of a central cylinder of silica surrounded with 10 to 50 concentric layers of biosilica (amorphous hydrated silica), which are separated from each other by thin and soft organic layers [37]. The anchor spicules have a diameter of around 50 µm and can reach a length of up to 10 cm. Due to the soft protein layers, potential cracks are not conducted directly through the material but are deflected between the individual layers. A study of the toughness, hardness and elastic modulus of the monolithic hydrated silica cylinder and the surrounding layered regions of hydrated silica and proteinaceous material by nanoindentation found a factor of 2.5 times higher toughness for the layered region. However, the monolithic cylinder displayed a 20% higher hardness and modulus [38]. With a tensile strength of 514 ± 178 MPa and a tensile modulus of 3800 ± 1400 MPa at a clamping length of 25 mm, the spicules [39] are admittedly less strong and stiff than conventional E-glass fibres with a strength of 2580 ± 40 MPa and a Young’s modulus of 78,000 MPa [40]. Nevertheless, due to the increased crack length, the bioglass fibres achieve a significantly higher toughness than conventional glass fibres [38,41,42,43]. The principle of crack propagation found in these bioglass fibres was applied to polypropylene (PP) sheets by layering talcum-filled PP as the stiffer component and unfilled PP layers as the softer component using the extrusion processing technique. It was found that the toughness of this material could be increased considerably, but the stiffness decreased significantly compared to the talcum-filled PP. It is hypothesised that using fewer but thicker layers of talcum-filled PP could maintain the stiffness [44].

The model of the spicules of the deep-sea glass sponge seems suitable and easy to implement to improve the toughness of brittle bast fibre-reinforced composites. For this purpose, thin cellulose acetate (CA) foils, representing the organic and ductile soft layers in the spicules, are placed between kenaf layers, representing the strong, stiff, and more brittle components. A bio-based epoxy was used as a matrix. The influence of different numbers of CA foil layers on the mechanical characteristics such as tensile, flexural and impact properties is investigated. Based on the aforementioned literature on the toughness improvement of brittle materials and the aim of transferring crack deflection mechanisms such as those found in the spicules of deep-sea glass sponges to kenaf fibre-reinforced composites in an abstracted form, we address the following research questions.

- Can thin and flexible CA foils be processed into composite materials?
- Can cracks be successfully redirected through the CA foils to increase toughness?
- How does the addition of CA foils change tensile and bending properties?

## 2. Materials and Methods

### 2.1. Materials

Kenaf fibre bundles (*Hibiscus cannabinus* L.) from Bangladesh with an average length of 30 to 40 mm supplied by Holstein Flachs (Mielsdorf, Germany) were used as reinforcing fibres. The fibres have not undergone any surface treatment and are used as supplied. The fibres were produced for applications in automotive interiors. The fibre properties were investigated in a previous study. The mean fibre (bundle) width measured with the FibreShape image analysis tool was 46.8 ± 24.9 µm. The mean tensile strength was determined with 653 ± 363.8 MPa and the tensile modulus with 26,800 ± 11,200 MPa at a clamping length of 3.2 mm and a test speed of 2 mm/min [45]. The matrix used was a bio-based epoxy resin type Greenpoxy 56 with an SD 7561 hardener (mixing ratio 100:36 by mass; density of 1.135 g/cm^3^) supplied by Sicomin (Châteauneuf Les Martigues, France). Matrix characteristics were determined in a previous study. The tensile characteristics of casted standard test specimens type 1A with a thickness of 4 mm were measured according to DIN EN 579-4 [46] with a test speed of 2 mm/min. The median tensile strength was determined with 61.0 ± 7.4 MPa, the tensile modulus with 3800 ± 700 MPa and the elongation at break with 2.2 ± 0.5%. The unnotched Charpy impact strength was analysed according to DIN EN ISO 179-1 [47] for 4 mm thick test specimens at a span length of 62.5 mm with 15.1 ± 6.1 kJ/m^2^. Cellulose acetate (CA) foil (Acetat Folie transparent type Ultraphan) with a thickness of 0.1 mm from Modulor (Berlin, Germany) was used as a type of impact modifier.

### 2.2. Composite Production of Kenaf/CA Hybrid Composites

**Figure 1:**
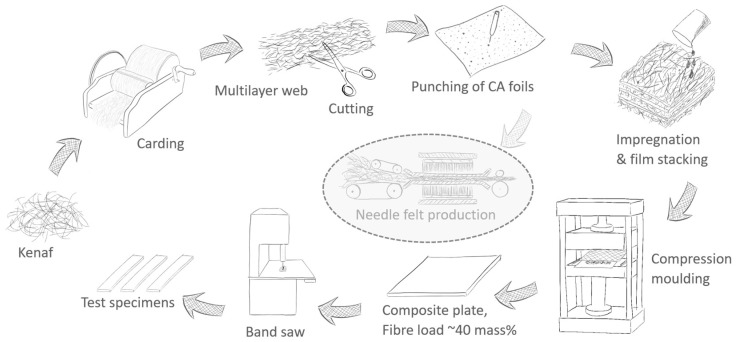
Production of kenaf fibre-reinforced composites with CA foils as an impact modifier. Optionally, the possibility for further processing the multilayer webs into needle felts as semi-finished products is shown in light grey.

The general process of manufacturing the composites inspired by the spicules of the deep-sea glass sponge is shown in Figure 1. In the first step, multilayer webs were produced with a carding machine. For this purpose, kenaf fibre bundles were carded three times using a manual roller card (Standard 46 tpi, size 78 × 19 cm^2^, Louët, Lochem, The Netherlands). According to [48], bast fibres for producing nonwoven like fleeces and needle felts should have a length of at least 60 mm. The cut length of the kenaf fibres used here is shorter, averaging 30–40 mm. However, longer fibre bundles are also included, since not all fibres were completely parallelised during the cutting process. In addition, the fibre bundles are opened during the carding process, resulting in single fibres and fine fibre bundles being pulled out of the coarser bundles. Due to the formation of fine single fibres and fibre bundles, the multilayer fibre web is held together as a semi-finished product. The multilayer webs were then cut to a size of approx. 25 × 19 cm^2^ and dried at 60 °C in an oven for at least 18 h (Universal oven UN 450, Memmert GmbH + Co.KG, Schwabach, Germany). The mass of the multilayer fibre web was based on the dry mass and was calculated to achieve a fibre mass proportion of 40% for a target plate thickness of 2 mm.

CA foils were cut to the same dimensions as the kenaf multilayer webs and punched manually with a needle to create small holes (~8–10 holes/cm^2^) to allow the matrix to penetrate between the foils and the fibres. The dried multilayer webs, representing the amorphous hydrated silica layers in the spicules, were then layered with CA foils, which are used as soft layers in an abstracted form for the protein-like layers of the spicules. A kenaf sample without CA foil was used as a reference sample. To investigate the influence of the CA foils, kenaf multilayer webs and a different number of CA foils were used (1, 3, 4 or 5 CA foils). The CA foils were distributed homogeneously between the kenaf layers, whereby the outer layers were always kenaf. All layers were individually impregnated with the Greenpoxy matrix (GP) and stacked. Subsequently, a cold compression moulding process was carried out at room temperature with a workshop press (Unicraft WPP 10 TE, Hallstadt, Germany) between two steel plates at a pressure of 10 tonnes. In order to maintain the plate thickness, two 2 mm thick aluminium spacers were positioned around the prepared sample. After 24 h, the plates were demoulded and annealed at 60 °C in the oven for 18 h, as the manufacturer’s data sheet recommended, to ensure complete cross-linking of the resin. The plates were sawn with a band saw (type MBS 240/E Proxxon, Föhren, Germany) into the appropriate specimen geometry for tensile, bending and impact tests and ground with sandpaper before use. In order to avoid delamination during the sawing process, fine-toothed saw blades suitable for the preparation of composite materials were selected (type PROXXON 28176, Wecker, Luxembourg).

### 2.3. Composite Production of Hybrid Composites Containing Different Kinds of Fibres

In order to compare how effective the improvement in toughness (unnotched Charpy impact strength) of the kenaf/CA foil hybrid composites is compared to hybrid composites made of different fibres, various hybrid materials were produced in four series of experiments using different processes.

1. Kenaf and kenaf/lyocell-reinforced PLA composites were produced by compression moulding. The kenaf fibre bundles and lyocell fibres with a fineness of 3.3 dtex and a length of 60 mm (Tencel, Lenzing AG, Lenzing, Austria) were used for the composite materials. The PLA matrix was used in the form of Ingeo fibres type SLN 2660 D; Eastern Textile Ltd., Taipei, Taiwan) with a fineness of 6.7 dtex and a staple fibre length of 64 mm produced from a NatureWorks^TM^ 6202D PLA. The reinforcing fibres were added with a fibre mass fraction of 40% to 60% PLA fibres. Pure kenaf and lyocell composites, as well as hybrid materials consisting of 20% kenaf and 20% lyocell in a homogeneous mixture as well as a layered hybrid material composed of 20% kenaf and 20% lyocell, in which two outer kenaf layers enclosed a lyocell core layer, were produced. The reinforcing fibres and the PLA fibres were blended into multilayer fibre webs with a preferred fibre orientation in the longitudinal direction to the production direction using a carding process. Further processing into composite plates with a thickness of approximately 2 mm was carried out using a compression moulding process described in a previous study [49].
2. Kenaf and kenaf/lyocell-reinforced PLA composites were produced by injection moulding. For this purpose, the kenaf, lyocell and PLA fibres, as described under process (1.) were used. Pure kenaf and lyocell composites with a fibre mass content of 40% and a homogeneous mixture of 20% kenaf and 20% lyocell fibres with a thickness of 4 mm were produced by injection moulding. The procedure for test specimen production was described previously [49].
3. Flax, PLA, aramid, flax/PLA and flax/aramid fibre-reinforced epoxy composites were produced by pultrusion. A Lincore flax fibre roving type FR 500 with a fineness of 500 tex (Depestele, Bourguébus, France), an aramid fibre roving with a fineness of 850 tex (Conrad Electronic SE, Hirschau, Germany), and a PLA fibre roving with a fineness of 100 dtex, type f 64 glz rd (Trevira GmbH, Bobingen, Germany) were used for the production of unidirectional-reinforced pultruded rectangular rods with a thickness of approx. 4 mm and a fibre volume content of 30%. A bio-based epoxy resin was used as the matrix (type SR GreenPoxy 56 resin with hardener SD 7561, mixing ratio 100:36 by mass; Sicomin, Châteauneuf Les Martigues, France). The test specimens were manufactured as described in [13].
4. Flax, carbon and flax/carbon-reinforced epoxy composites were produced by cold compression moulding. For the production of approx. 2 mm thick composite laminates with a surface area of 20 × 20 cm^2^, a flax fabric of the type Amplitex 5040 with a mass per unit area of 300 g/m^2^ with a twill weave from the company Bcomp Ltd. (Fribourg, Switzerland) and a carbon fibre fabric (HT carbon fibre) with a mass per unit area of 200 g/m^2^ with a plain weave from R&G Faserverbundwerkstoffe (Waldenbuch, Germany) were used. An epoxy resin type Epikure RIMR135 with a hardener type RIMH137 (mixing ratio 100:30 by mass) from Lange + Ritter GmbH (Gerlingen, Germany) was used as the matrix. A carbon fibre-reinforced epoxy made of 6 layers of carbon fabric and a composite material made of 3 layers of flax fabric (predried for 2 h at 101 °C in a forced air oven) were produced. This results in a fibre volume content of 36% for the carbon fibre-reinforced plastic and 33% for the flax fibre-reinforced composite. A hybrid material was produced from two layers of carbon, which were used as outer layers, and a core of two layers of flax, resulting in a fibre volume content of 13% carbon and 24% flax. A silicone frame with a thickness of 2 mm and an inner cut-out area of approx. 22 × 22 cm^2^ was placed on a vacuum foil, which was placed on the lower press plate made of steel. The fabric layers were placed in the silicone frame and coated manually with the epoxy resin layer by layer. The vacuum foil was then wrapped, and the press plate was placed in the workshop press (Unicraft WPP 10 TE, Hallstadt, Germany); two steel spacers of 2 mm thickness were placed around the silicone mould underneath the vacuum foil, and the setup was covered with a second steel press plate. A pressure of 5 tonnes was applied for the pressing process. After curing, the plates were removed, and test specimens were cut out for characterisation.

The nonreinforced matrices were also examined in all four test series to determine whether the fibres had a reinforcing effect.

### 2.4. Testing

Before testing, the CA foils and the composites were conditioned for at least 18 h at 23 °C and 50% relative humidity according to DIN EN ISO 291 [46]. The CA foils were characterised using tensile tests. For this purpose, 10 test specimens with type 1A geometry according to DIN EN ISO 527-4 [46] were produced with the help of a metal template and a cutter knife. The measured average thickness of the foils was 0.11 ± 0.01 mm. The test was performed at a clamping length of 100 mm and a test speed of 10 mm/min with a universal testing machine type Z020 (Zwick/Roell GmbH, Ulm, Germany) equipped with a 500 N load cell and a manual clamping system (type 8135/20 N, metal coating). The strain was recorded via the traverse path of the machine, and Young’s modulus was determined between 0.05 and 0.25% deformation.

The tensile properties of the kenaf and kenaf/CA composites produced were also determined with the universal testing machine Z020 equipped with a 20 kN load cell (Zwick/Roell GmbH, Ulm, Germany) and a pneumatic clamping system (clamping pressure 1.5 to 2.0 bar). The tensile properties of rectangular test specimens with dimensions of 250 × 25 mm^2^ were determined on at least 5 test specimens per variant according to DIN EN ISO 527-4 [46] with a clamping length of 100 mm after reaching a preload of 1 MPa at a test speed of 2 mm/min. The strain for determining Young’s modulus in a range between 0.05 and 0.25% deformation was carried out with a video extensometer (Zwick VideoXtens, Ulm, Germany) between two measuring marks spaced 50 mm apart on the specimen.

The bending properties of kenaf and kenaf/CA composites of at least 5 test specimens per test series with dimensions of 80 × 15 mm^2^ were investigated with a three-point bending test. For this purpose, the universal testing machine was equipped with a load applicator (radius of 5 mm) and supports (radius of 2 mm). The support distance was set to 16 × h (h = specimen thickness) in accordance with the DIN EN ISO 14125 standard [50]. The test started after reaching a preload of 10 N. The test speed was 1 mm/min to determine the bending modulus between 0.05 and 0.25% bending strain and then accelerated to 5 mm/min until breakage of the specimen. The bending strain was calculated from the traverse path of the machine.

The unnotched Charpy impact strength of kenaf, kenaf/CA and hybrid composites was measured on 6 test specimens per test series with the dimensions of 80 × 10 mm^2^. A Zwick pendulum impact tester, type 5102 (Zwick/Roell GmbH, Ulm, Germany), equipped with a 2 J or 4 J pendulum hammer, was used. The bearing distance was set according to the standard DIN EN ISO 179-1 [47], resulting from the specimen thickness h multiplied by the factor 20.

### 2.5. Scanning Electron Microscopy (SEM) Investigation

SEM analyses of composite fracture surfaces were performed with a SEM type JSM 6510 (Jeol, Eching, Germany) operating with emission electrons. Before the observation, the samples were sputtered with a layer of platin–palladium with a Cressington Sputter coater (Dortmund, Germany), working with a current of 25 mA for 100 s. The SEM investigations were conducted with an acceleration voltage of 10 kV.

### 2.6. Evaluation

The evaluation was performed with the open-source software R Studio, version 1.4.1103/R version 4.0.3. The results were examined for normal distribution with a Shapiro–Wilk test. The homogeneity of the variances was examined with a Levene test. Since not all results were normally distributed or the variances of the different results were not homogeneous, a Wilcoxon test was then used to compare the median values of all test results. All tests were performed with a probability of error α = 5%. The composites’ results are shown as bar plots with the mean arithmetic deviation (MAD) as error bars. Not normally distributed results are marked with an asterisk, and significant differences are labelled with different letters.

## 3. Results & Discussion

### 3.1. Tensile and Bending Characteristics

**Figure 2:**
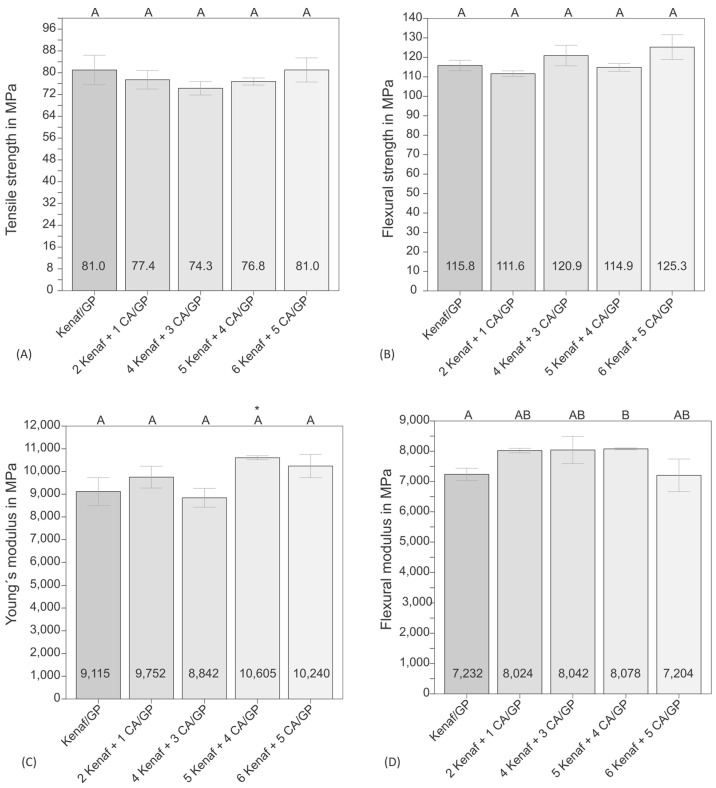
Tensile strength (**A**), flexural strength (**B**), Young’s modulus (**C**) and flexural modulus (**D**) of the kenaf composite and hybrid materials with different numbers of CA foil layers. The bars represent the median values, and the error bars represent the mean arithmetic deviation (MAD). An asterisk * indicates results that are not normally distributed; different letters represent significant differences.

**Figure 3:**
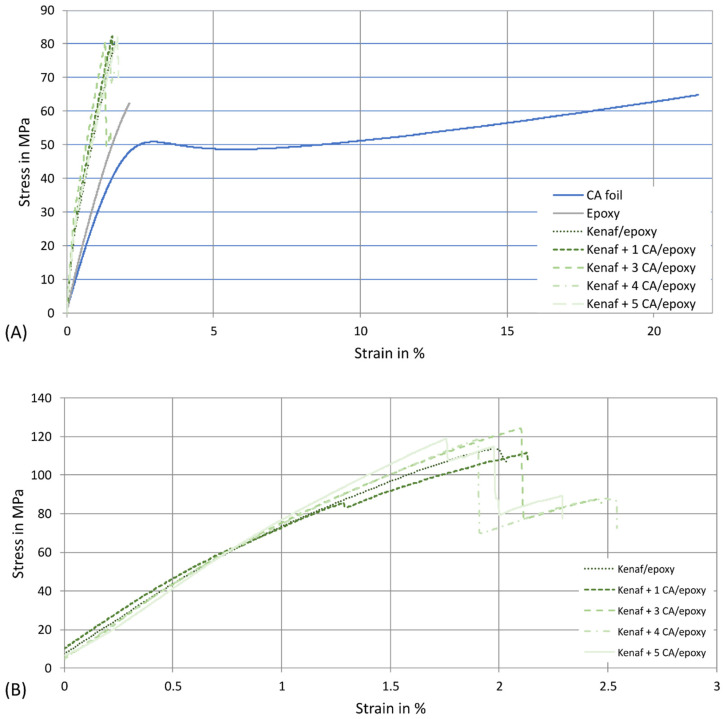
Stress-strain curves from the tensile tests (**A**) and the bending tests (**B**) of different materials.

The results of the tensile and bending tests demonstrate that inserting CA foils between the multilayer fibre webs does not affect these composite properties significantly. The tensile strength (Figure 2A) could be increased for all materials to 74 to 81 MPa compared to the pure Greenpoxy matrix with a value of 61.0 ± 7.4 MPa. The presence of CA foils does not significantly affect the tensile strength. The strength of the CA foils was determined to be 64.9 ± 6.1 MPa and is, thus, in the same range as the value of the Greenpoxy matrix. If the rule of mixtures [51] is applied, a comparable value would also be calculated for all materials since the material thickness is kept constant and the CA foils replace part of the matrix. Figure 3A shows the stress–strain curves of the tested materials. It can be seen that the kenaf-reinforced materials achieve lower strain values than the epoxy matrix. The CA foils achieve a similar maximum stress as the epoxy matrix but have a completely different stress–strain behaviour with a significantly higher elongation. This aspect significantly influences the toughness and will be discussed later. As the failure of the kenaf materials in the tensile test is essentially based on the weakest link theory [52], the CA foils only lead to a slight increase in elongation at break compared to the kenaf material without CA foils. The tensile strength of the kenaf/Greenpoxy composites is clearly higher compared to 40 mass% kenaf-reinforced PLA composites with a tensile strength of 53 MPa determined in a previous study [53] and in the same range of 40 mass% kenaf-reinforced PLA manufactured with improved compression moulding process parameters achieving a value of 82 MPa [45].

The bending strength results (Figure 2B) show that the CA foils also have no significant influences on the composite properties. The values of the bending strength are considerably higher than the tensile strength values, ranging between 112 and 125 MPa, which can be justified by the stress–strain behaviour of the materials. Strictly speaking, bending tests should only be used for materials that behave linearly elastically up to failure. This was not the case for the composites tested. By leaving the linear elastic region at a particular force value, the elasticity limit is initially exceeded at the edge of the sample, whereby plastics or fibre-reinforced plastics can absorb excessive loads for a short time. In this case, the bending strength is overestimated, leading to higher values [54]. This phenomenon was also observed, e.g., for bio-based materials based on polyhydroxyalkanoates (PHA) [55,56]. The stress-deformation curves from the bending test are shown in Figure 3B. It is apparent from the graph that the composite materials produced do not exhibit strictly linear elastic behaviour from the start to the end of the test. However, the behaviour of the materials with CA foils differs from that of the pure kenaf composite. The CA foils lead to delamination, which prevents spontaneous total failure of the structure. While the pure kenaf composite material failed spontaneously and was brittle, a layer-like failure occurred in the materials with CA foils, thus delaying the failure of the entire structure. The deformation of the materials up to complete failure could thus be delayed, which is an essential aspect of the increase in toughness discussed later. The flexural strength of the kenaf and kenaf/CA composites is in the same range as 40 mass% kenaf-reinforced PLA with a value of 126 MPa investigated in a former study [45].

Young’s modulus from the tensile test in Figure 2C shows a scatter of the measurement results in the range between 9100 and 10,600 MPa, which, however, do not differ significantly from each other from a statistical point of view. Compared to the neat matrix with a Young’s modulus of 3870 ± 660 MPa, all composites show significantly higher values and the fibres, thus, have a clear reinforcing effect. The pure CA foils resulted in a lower tensile modulus of 2904 ± 200 MPa. Consequently, the tensile modulus should decrease with more CA layers. Since this is not the case, it is assumed that determining the tensile modulus via the traverse path instead of using the video extensometer on the one hand and that CA is used in the form of foils, on the other hand, plays a role. It has been shown that the tensile modulus is somewhat lower when measured via the traverse path, as the compliance of the testing machine is excluded using an extensometer. The example of PLA has shown that foils (thickness ~50 to 200 µm) can have lower Young’s moduli than compact specimens with a thickness ≥2 mm (unpublished results). Therefore, it cannot be excluded that Young’s modulus of epoxy would be lower when measured on thin resin foil. In the same way, Young’s modulus could be higher if it is determined on compact CA samples instead of thin foils. Finally, it was shown that the CA foils do not significantly influence Young´s modulus of the composites, leading to the assumption that the properties of the CA foils and the matrix must be similar. Kenaf fibre-reinforced PLA composites investigated in a former study resulted in a lower Young´s modulus of 7600 MPa [45].

The flexural modulus results (Figure 2D) also show a larger scatter and range between 7200 and 8100 MPa. The composite made of kenaf with four layers of CA foils differs significantly from the reference sample made of kenaf. All other samples do not vary significantly. Since the characteristic value of the sample with four CA layers is very similar to the values of the sample with one CA layer and three CA layers, it is assumed that the difference is to be found in the very low standard deviation. The results of the flexural modulus are generally lower than those of the tensile modulus. This effect is described by Jaroschek [54] and is justified by the different load applications during bending and tensile tests. It has been shown that the modulus of elasticity from the tensile test of polymeric materials is often approx. 5% higher than from the bending test [54]. A similar effect was also observed, e.g., for bio-based materials based on polyhydroxyalkanoates (PHA) [55,56]. Compared to results measured for 40 mass% kenaf-reinforced PLA resulting in a flexural modulus of 6900 MPa, the values of the presented kenaf/Greenpoxy composites are clearly higher [45].

The elongation at break determined by the tensile test ranged between 1.5 and 1.7% for the kenaf composites and the composites modified with CA foils. The Greenpoxy matrix achieved values of 2.2% and the CA foils of 20.3% (see Figure 3A), meaning that the CA foils have significantly higher ductility than the kenaf layers, similar to the tough protein layers compared to the brittle bioglass layers in the biological role model (spicules). Due to the brittle nature of kenaf and the weakest link theory [52], the elongation at break of the composites was significantly reduced compared to the matrix, as already described in the literature for bast fibre-reinforced composites [57]. Under bending load (Figure 3B), the presence of the CA foils increases deformation and prevents spontaneous failure of the composite structure. In some cases, the individual layers failed, which can be seen in the stress-train curves by the step-like drop in force.

In the whole consideration, it can be concluded that the presence of CA foils does not significantly affect the tensile and flexural strength and modulus values of the kenaf-reinforced composites.

### 3.2. Toughness

**Figure 4:**
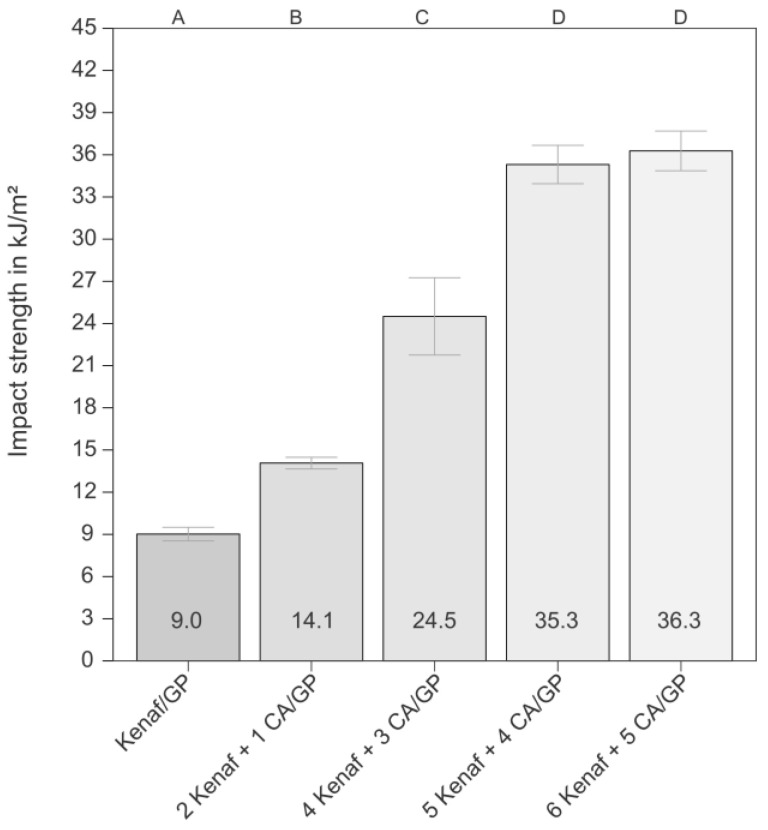
Unnotched Charpy impact strength of the kenaf composite and hybrid materials with different numbers of CA foil layers. The bars represent the median values, and the error bars represent the mean arithmetic deviation (MAD). All results are normally distributed; other letters represent significant differences.

In contrast to the tensile and bending properties, the work at break and the unnotched Charpy impact strength were influenced by the CA foils. The work at break determined with the tensile tests increased only slightly from 2878 ± 329 Nmm for the kenaf/GP composite to 3228 ± 454 Nmm for the kenaf/GP composite with five CA foils while the impact strength was significantly affected. The impact strength of the pure kenaf/GP sample, shown in Figure 4, is below the impact strength value of the pure matrix (15.1 ± 6.1 kJ/m^2^). A former study reported a similar result for 40 mass% kenaf-reinforced PLA, where the matrix achieved an impact strength of 17 kJ/m^2^ and the composite of 14 kJ/m^2^ [45]. When using one CA foil, the impact strength was significantly improved compared to the kenaf/GP composite. Using three CA foils, the impact strength could be increased significantly above the value of the pure matrix and with four CA foils, a further significant increase is achieved. This represents an increase in impact strength by a factor of 2.3 compared to the pure matrix and a factor of 3.9 compared to the kenaf/GP sample. Only a slight increase in impact strength was observed from the use of four foils onwards.

**Figure 5:**
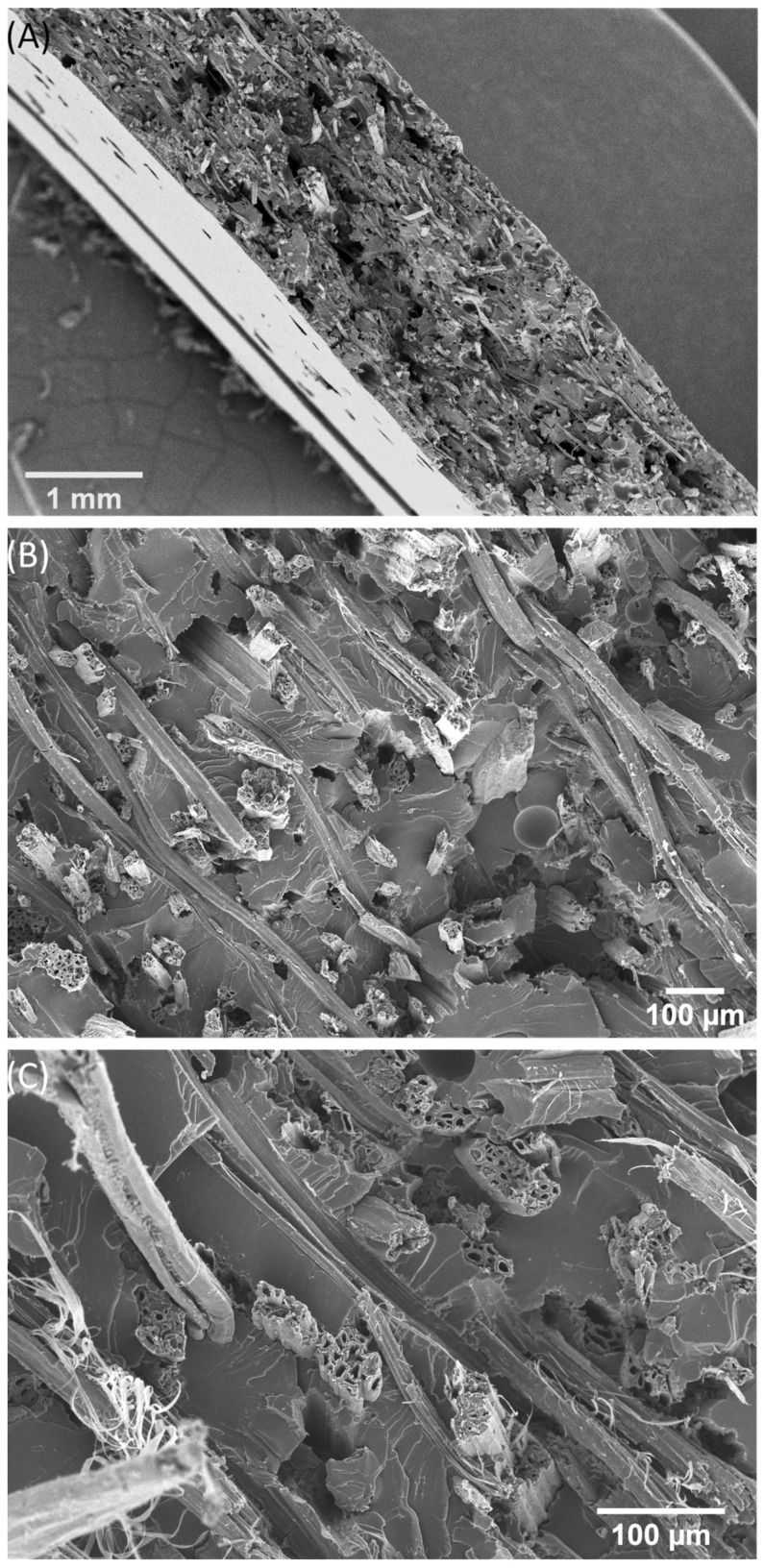
SEM micrographs of the fracture surfaces from the tensile test of a kenaf-reinforced composite. (**A**) overview, (**B**,**C**) detailed images.

Following a natural role model, the aim of improving the toughness of brittle bast fibre-reinforced composites without negatively influencing other properties, such as tensile or bending properties, was thus achieved. The main question was, “Why was it possible to significantly improve the toughness of the materials using CA foils?” The answer to this question is found in the fracture behaviour of the materials, which is similar to the fracture behaviour of the spicules of the deep-sea glass sponge. SEM analyses of the fracture surfaces of kenaf/GP composites and composites made of kenaf/GP with four CA foils were carried out and analysed. Figure 5 shows the fracture surface of a kenaf/GP composite. The overview micrograph in Figure 5A displays the brittle fracture behaviour typical for bast fibre-reinforced composites. It becomes evident that there are also some voids in the sample volume. However, the detailed micrographs in Figure 5B and C show relatively good adhesion between the fibres and the matrix. There are hardly any or only tiny gaps between the fibre and the matrix, so a good load transfer can be assumed from the matrix to the fibre. However, the pull-out lengths of the fibre bundles are relatively short (Figure 5B), which means that only little energy is absorbed during fracture, as energy-absorbing long fibre pull-outs leading to higher toughness are not available. This effect has already been described extensively in the literature [2]. Bast fibres, combined with polymer matrices, usually lead to an increase in stiffness and frequently also in strength, whereby the toughness of the matrix is often not achieved.

**Figure 6:**
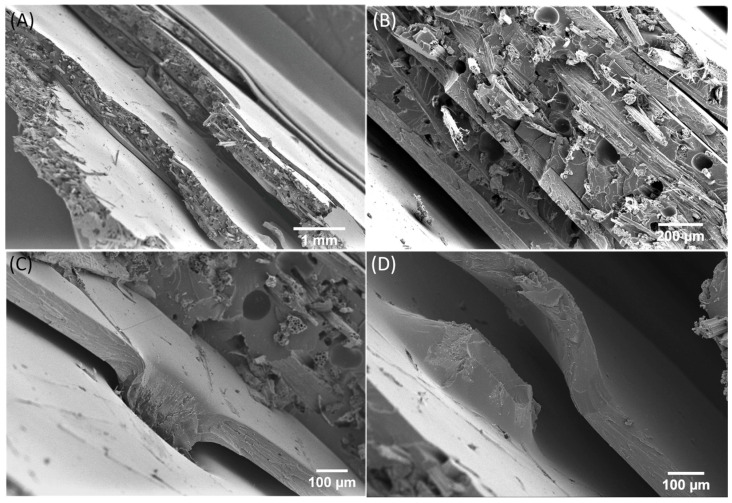
SEM images of the fracture surfaces from the tensile test of a kenaf/CA foil hybrid composite with four layers of CA foils. (**A**,**B**) overview, (**C**,**D**) detail images of the connection between the punched CA foils and the kenaf multilayer webs.

**Figure 7:**
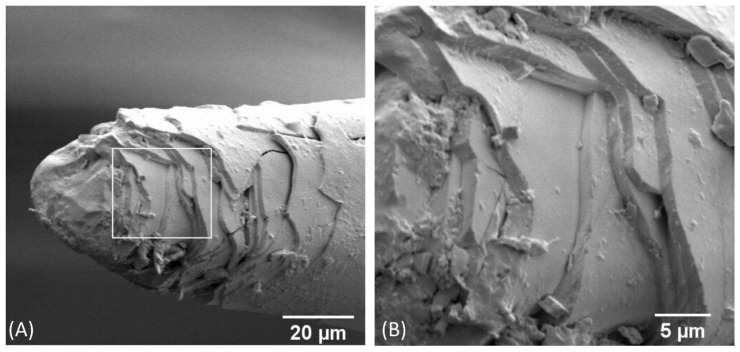
SEM micrographs of the fracture surface of a deep-sea glass sponge (Hexactinellida) anchor spicule, (**A**) overview, (**B**) detailed micrograph from the marked region in (**A**).

The fracture behaviour of the CA foil samples (Figure 6) differs significantly from the kenaf/GP reference sample in Figure 5. In the overview in Figure 6A, a gradual fracture is visible, similar to the fracture surface of the bioglass fibre in Figure 7. The individual kenaf layers separated by the CA foils show fractures on different planes. This behaviour could already be observed when the materials failed in the three-point bending test (Figure 3B). In this case, it was shown that the CA foils led to a delay in failure under bending load compared to the pure kenaf sample. A closer view in Figure 6B shows that the fracture behaviour of the individual kenaf layers is very similar to that of the kenaf/GP reference sample. The fibres show low pull-out lengths, and the fracture behaviour of the material appears brittle. Due to the perforation of the foils with small holes, the foils partially show a stronger adhesion where the matrix could penetrate through the holes and a weaker adhesion in places that were not perforated. This effect can be seen in Figure 6A,B. In some areas, the foils appear to be well bonded to the kenaf layers; in others, there are gaps between the foils and the kenaf layers. Figure 5C shows how the bonding of the CA foils and the kenaf layers works. At the location of the hole in the foil, there seems to be a good bond, whereas, in the neighbouring area, a detachment of the foil from the kenaf layer can be found. Figure 6D shows a complete debonding of the foil from the kenaf layer due to the breakage of the specimen. Compared with the abstracted role model of the spicules of the deep-sea glass sponge, the fracture behaviour between the kenaf and CA layers is similar to the spicules (Figure 7). When the spicules fail, delamination between biosilica and the thin organic layers, leading to noncatastrophic failure, is also reported [39].

It is assumed that the CA foils lead to fracture branching and crack deflection, as shown in Figure 6A. Crack deflection changes the fracture path, and more energy is absorbed than the crack’s direct passage through the specimen. The increased energy absorption improves the toughness of the materials. The more foils are used, the more energy is absorbed by the crack deflection and the impact strength increases, whereby it has been shown that with the material pairing investigated here and a specimen thickness of approx. 2 mm, only minor improvements can be achieved with a higher number of four foils.

Miserez et al. [38] investigated the differences in fracture toughness between the monolithic hydrated silica cylinder and the surrounding layered regions of hydrated silica and proteinaceous material in spicules using nanoindentation. The results showed that the fracture toughness of the layered regions is higher by a factor of 2.5 than that of the monolithic cylinder, which has a 20% higher hardness and modulus. The present study demonstrated no stiffness reduction using CA foils in the composite. Miserez [38] determined the modulus of elasticity by nanoindentation on the different structures, not on the entire structure. It can be assumed that the indentation modulus would also show differences between CA foils and fibre layers as the CA foils have a significantly lower Young’s modulus than the kenaf/GP composites. Even if the implementation of the natural role model was very generic and the geometric ratios of the thicknesses of the different layers were not considered, a similar fracture behaviour could be achieved (compare Figure 7). As the protein layer in the deep-sea glass sponge led to crack deflection, this task could be taken over by the CA foils in the kenaf composite and contribute to an individual failure of the specific layers, thus significantly increasing the fracture toughness.

### 3.3. Effectiveness of CA Foils as Impact Modifiers Compared to Hybrid Composites Made of Different Fibres

As discussed, bast fibres can lead to good strength and stiffness in a composite but usually result in low toughness [2]. Therefore, some applications where higher toughness is required are still not feasible. Many studies focus on improving the toughness of fibre-reinforced composites using impact modifiers [9,10], tougher fibres [45,58,59,60], special laminates [61,62] or bio-inspired concepts [17,19,20,21]. Hybridisation with different fibres is often used to improve certain composite material properties. For example, bast and glass fibres or bast and carbon fibres are used to combine the positive properties of both fibre types. For example, the combination of bast and glass fibres significantly improves toughness compared to pure bast fibre-reinforced composites [11,12,13,14]. A combination of bast and carbon fibres can lead to an increase in the stiffness and toughness of pure bast fibre-reinforced composites and, at the same time, significantly improve the damping properties of pure carbon fibre-reinforced composites [63,64,65,66]. It has also been shown that the fatigue strength of, e.g., flax fibre-reinforced materials is better than that of glass fibre-reinforced composites above a certain number of loading cycles [67]. However, other cellulose-based fibres can also be combined, e.g., bast and regenerated cellulose fibres, to increase the toughness of the bast fibre-reinforced composites [45,60].

**Figure 8:**
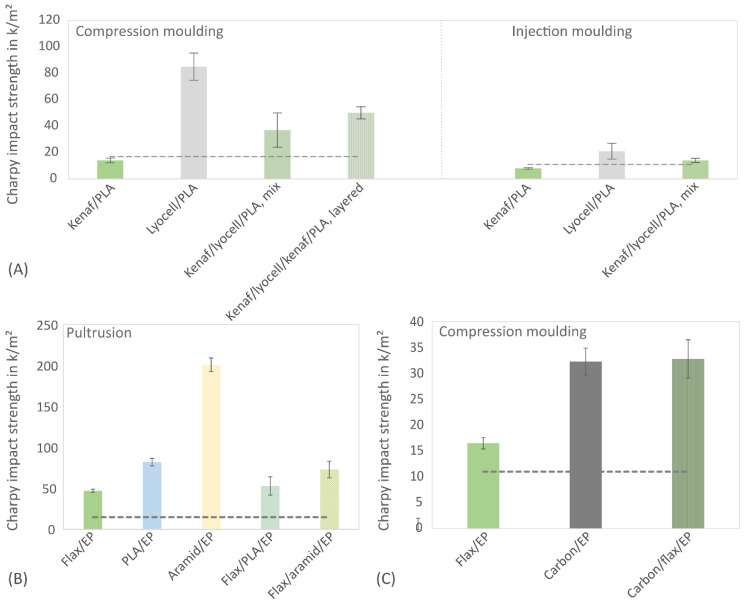
Unnotched Charpy impact strength (mean ± standard deviation) for different bast fibre-reinforced composites and hybrid materials. The dotted line represents the values of the pure matrix. (**A**) kenaf, lyocell and hybrid materials made of kenaf and lyocell produced in compression moulding and injection moulding process with a PLA matrix, (**B**) unidirectionally reinforced flax, PLA, aramid and hybrid materials with a bio-based epoxy matrix (Greenpoxy) produced using pultrusion process and (**C**) flax, carbon and hybrid materials made of fabrics in an epoxy matrix produced using compression moulding process.

The effectiveness of toughness improvement (unnotched Charpy impact strength) by the bio-inspired approach using CA foils is compared to hybrid composites consisting of bast and other fibres manufactured with different production processes (Figure 8). The dashed lines represent the characteristic values of the pure matrix. In Figure 8A, composites of kenaf, lyocell and a mixture of both were produced with a PLA matrix by compression and injection moulding (fibre mass fraction ~40%). Multilayer webs made of staple fibres with a fibre orientation predominantly in the longitudinal direction of the composite were used as the semi-finished products for the compression moulding process. In the injection moulding process, the fibres were present as short fibres with a random orientation. Figure 8B shows unidirectionally reinforced, pultruded composites of flax, PLA, aramid and a mixture of flax/PLA and flax/aramid in combination with a bio-based epoxy matrix. The fibre volume fraction was approx. 30%. Figure 8C represents the results of flax, carbon and flax/carbon composites produced by compression moulding with an epoxy matrix from woven fabrics with a fibre volume fraction of approx. 35%.

Figure 8A shows that the kenaf fibres do not improve the impact strength of the PLA matrix. Instead, lyocell fibres (regenerated cellulose) lead due to their high toughness to multiple higher impact strength values. Lyocell fibres are obtained in a so-called NMMO process [68] and can be obtained from various raw materials such as dissolving pulp, bleached softwood kraft pulp or sugar cane bagasse [69]. Production is also possible from bast fibres such as kenaf [69], flax [70] or hemp bast fibres [71]. In this way, brittle bast fibres can be converted into more ductile regenerated cellulose fibres. Compared to bast staple fibres, which are, e.g., used in the automotive industry, the production effort and costs of regenerated cellulose are significantly higher, which is why bast fibres such as kenaf continue to have a large field of application. As shown in Figure 8A, a homogeneous mixture of kenaf and lyocell (ratio 50:50) produced in the compression or injection moulding process results in a toughness increase of 164% and 163%, respectively, compared to the kenaf/PLA reference sample (compare Table 1). When a specific layering of kenaf and lyocell (ratio 50:50) similar to the bio-inspired composite following the structure of the coconut pericarp presented in [21] is applied, the impact strength is further increased by 257%. This result indicates the importance of selective layering of stiff outer layers and tougher inner layers of a material (in this case, stiffer kenaf layers as outer layers enclosing a tougher inner core of lyocell). Figure 8B summarises the results of unidirectionally reinforced materials; toughness was improved compared to the pure epoxy matrix, which was achieved with all fibres, including the flax bast fibre due to unidirectional fibre orientation. The highest impact strength could be achieved with aramid fibres, followed by PLA fibres. A blend of flax and PLA fibres or flax and aramid fibres (50:50 ratio) resulted in an improvement of 13% and 55%, respectively (compare Table 1). This increase is relatively small compared to a targeted layer build-up, as shown in Figure 8A for lyocell and kenaf. An effective influence of the layer structure is also shown in Figure 8C. The flax and carbon fabrics resulted in higher toughness than the epoxy matrix used, with the carbon fabrics resulting in twice the impact strength of the flax fabrics. For hybridisation, outer carbon layers surrounding a flax core were used. The fibre volume content of the carbon layers in this composite was only 13%, whereas the flax content was 24%. Despite the low carbon fibre content, the toughness of the hybrid material was increased by 99% compared to the flax fibre composite thanks to the targeted layered structure, and, thus, it almost corresponds to the toughness of the pure carbon fibre laminate.

Overall, the alternative approach using the bio-inspired structures of the deep-sea glass sponge spicules leads to the highest toughness improvement (see Table 1). The best results were obtained for the kenaf composites with four or five CA foils.

## 4. Conclusions

Based on our results, we can answer the research questions posed in the introduction as follows:

- CA foils have been successfully implemented between kenaf multilayer webs in a composite material.
- The CA foils, which imitated the protein layers in the biological role model, considerably changed the fracture behaviour of the composite material. The CA foils prevented a direct propagation of the crack under mechanical load, led to a crack deflection and, thus, increased the crack length and increase the fracture toughness. This phenomenon improved the unnotched Charpy impact strength by increasing the number of CA foils from one to five foils by up to 302% compared to the neat kenaf-reinforced composite.
- The CA foils did not negatively affect mechanical characteristics like tensile or bending properties. The approach described is explicitly based on improving the toughness of composite materials reinforced with bast fibre bundles.

It was found that the strength values of CA foils and the epoxy matrix used were similar. Therefore, the following should be further investigated:

- How foil types or matrices with different mechanical properties perform in the described composite structure;
- How effectively the approach can be transferred to composites made of bast fibre semi-finished products of higher quality (e.g., fabrics and rovings), which, in principle, lead to higher mechanical composite properties.

The process could be transferred to industrial applications as follows:

- The multilayer webs should be co-needled with the CA foils as semi-finished products for thermoset composite applications. For this purpose, the needle felt process could be implemented in the production process, as shown in Figure 1. Needling would automatically punch the foils, and fibres and fibre bundles would also protrude through the CA foils in the z-direction. It is assumed that the toughness could be further increased by better adhesion between the fibre and foil layers, and the manufactured materials could thus withstand higher impact loads. Incorporating or co-needling CA foils in thermosetting composite applications could contribute to use needle felts made of bast fibres in applications where a higher toughness is required.

## Figures and Tables

**Table 1:** Improvement of the unnotched Charpy impact strength of bast fibre-reinforced composites by hybridisation with CA foils or other fibre types. The improvement factor refers to the percentage increase in impact strength due to hybridisation compared to the reference samples reinforced only with bast fibre bundles.

Reinforcement	Matrix	Manufacturing Process	Improvement in %
Kenaf/1 layer CA	Greenpoxy	Compression moulding	56
Kenaf/3 layer CA	Greenpoxy	Compression moulding	172
Kenaf/4 layer CA	Greenpoxy	Compression moulding	291
Kenaf/5 layer CA	Greenpoxy	Compression moulding	302
Kenaf/lyocell, mix	PLA	Compression moulding	164
Kenaf/lyocell/kenaf, layered	PLA	Compression moulding	257
Kenaf/lyocell, mix	PLA	Injection moulding	163
Carbon/flax/carbon, layered	Epoxy	Compression moulding	99
Flax/PLA, mix	Epoxy	Pultrusion	13
Flax/aramid, mix	Epoxy	Pultrusion	55

## Data Availability

The data can be made available on request.
